# Abundant Parent-of-origin Effect eQTL: The Framingham Heart Study

**DOI:** 10.1101/2024.06.05.597677

**Published:** 2025-06-04

**Authors:** Yongtao Guan, Tianxiao Huan, Daniel Levy

**Affiliations:** National Heart, Lung, and Blood Institute

**Keywords:** parent-of-origin effect (POE), expression quantitative trait loci (eQTL), paternal eQTL, maternal eQTL, opposing eQTL, Bayes factors

## Abstract

Parent-of-origin effect (POE) is a phenomenon whereby an allele’s effect on a phenotype depends both on its allelic identity and parent from whom the allele is inherited, as exemplified by the polar overdominance in the ovine callypyge locus and the human obesity *DLK1* locus. Systematic studies of POE of expression quantitative trait loci (eQTL) are lacking. In this study we use trios among participants in the Framingham Heart Study to examine to what extend POE exists for gene expression of whole blood using whole genome sequencing and RNA sequencing. For each gene and the SNPs in cis, we performed eQTL analysis using genotype, paternal, maternal, and joint models, where the genotype model enforces the identical effect sizes on paternal and maternal alleles, and the joint model allows them to have different effect sizes. We compared models using Bayes factors to identify paternal, maternal, and opposing eQTL, where paternal and maternal effects have opposite directions. The resultant variants are collectively called POE eQTL. The highlights of our study include: 1) There are more than 2, 000 genes harbor POE eQTL and majority POE eQTL are not in the vicinity of known imprinted genes; 2) Among 180 genes harboring opposing eQTL, 99 harbor exclusively opposing eQTL, and 58 of the 99 are phosphoprotein coding genes, reflecting significant enrichment; 3) Paternal eQTL are enriched with GWAS hits, and genes harboring paternal eQTL are enriched with drug targets. Our study demonstrates the abundance of POE in gene expression, illustrates the complexity of gene expression regulation, and provides a resource that is complementary to existing resources such as GTEx. We revisited two previous POE findings in light of our POE results. A SNP residing in *KCNQ1* that is maternally associated with diabetes is a maternal eQTL of *CDKN1C*, not *KCNQ1*. A SNP residing in *DLK1* that showed paternal polar overdominance for human obesity is a maternal eQTL of *MEG3*, offering an explanation for the baseline risk of homozygous samples through association between *MEG3* expression and obesity. Finally, we advised caution on conducting Mendelian randomization using gene expression as the exposure.

## 1 Background

Parent-of-origin effect (POE) is a phenomenon whereby an allele’s effect on a phenotype not only depends on its allelic identity, but also on the parent from whom the allele is inherited (DeChiara et al., 1991; Cockett et al., 1996). POE can be driven by factors such as sex bias in transmission of genetic variants (Tomé et al., 2011) and maternal genetic effects that influences the environment in which the offspring develops (Hager et al., 2008). The main driver, however, is genomic imprinting, which is a phenomenon where one of the two alleles at a locus is functionally silenced by methylation. Importantly, which parental copy of the allele being silenced is highly consistent for that gene across samples. Imprinting appears to play an important role in embryonic and placental development and social behavior (Reik and Walter, 2001; Garfield et al., 2011). Genes underpinning syndromic disorders such as Prader-Willi, Beckwtih-Weidemann, and Angelman show POE (Lawson et al., 2013). The same is also true for common disease phenotypes, such as breast cancer, diabetes, and cardiovascular disease (Kong et al., 2009; Hanson et al., 2013; Mozaffari et al., 2019).

In genetic association studies, particularly genome-wide association studies (GWAS), the absence of POE is usually assumed implicitly. This assumption is largely out of convenience, as in most GWAS, the parent-of-origin of an allele is not directly observable, and not inferable due to lack of first and second degree relatives in the sample. Exceptions include an Icelandic cohort, with their extensively documented pedigrees and the abundance of closely related samples (Kong et al., 2009), a Hutterite pedigree (Mozaffari et al., 2019), the Framingham Heart Study with three-generations of participants (Kannel et al., 1979), and the biobank datasets, for which parent-of-origin can be inferred using distant relatives (Hofmeister et al., 2022). Another contributing factor to the implicit assumption may be the presumed scarcity of documented POE in humans. Although POE is believed to be associated with a wide range of complex traits and diseases (Lawson et al., 2013), significant associations reported in GWAS setting are of limited quantity. For example, in a Hutterite study, Mozaffari et al. (2019) examined 21 phenotypes and produced 18 significant associations with POE, fewer than one association per phenotype.

Mouse studies demonstrated that, despite a limited number of imprinted genes, most phenotypes display POE, and loci that show POE were not enriched for known imprinted genes. Using reciprocal F1 crosses, Mott et al. (2014) showed that non-imprinted genes can generate POE by interactions with imprinted loci. Focusing on metabolic traits and using a mouse population at different levels of intercrossing, Macias-Velasco et al. (2022) identified a network comprised of three imprinted and six non-imprinted genes that show POE. In humans, however, loci with POE in relation to phenotypes are either near imprinted regions by design (Kong et al., 2009; Hanson et al., 2013), or near regions with characteristic of imprinting from a genome-wide scan (Mozaffari et al., 2019). To what extend POE contributes to phenotypic variation in humans and how widespread the loci with POE in the human genome remains elusive.

Using whole genome sequencing and RNA sequencing data from the Framingham Heart Study, and taking advantage of its relative abundance of trios, we set to investigate to what extent and degree gene expressions is affected by parent-of-origin. There exist multiple studies on POE on gene expression (Zhabotynsky et al., 2019; Jadhav et al., 2019; Deng et al., 2020) or POE of methylation and gene expression jointly (Zink et al., 2018), but these studies are either underpowered due to small sizes or focus on analysis of allele specific gene expression instead of eQTL. A systematic study of POE eQTL is lacking and our study aims to fill this void.

## 2 Results

### 2.1 Overview of data processing

We identified 1477 trios with whole genome sequencing (WGS) data and children within the trios having RNAseq data. After routine QC of genotype data, for each trio, we first phased the child based on rules of Mendelian inheritance, which resolved all non triple heterozygous genotypes. The genotype data has negligible Mendelian error rate, and see details in Methods on how to handle them.

We then marked the remaining triple heterozygous genotypes as missing, which produced phased paternal and maternal haplotypes for each child in the trio. Next we imputed missing alleles into their paternal and maternal haplotype background, and the parental haplotype with a higher imputed allele dosage was assigned the reference allele, the other haplotype was assigned the alternative allele. Simulation studies showed this mask and impute phasing to be highly accurate (Supplementary Table S1). Thus at each SNP, we have a genotype vector, a paternal vector, and a maternal vector.

To process RNAseq data, we started from Transcript Per Million (TPM) values, and selected 16, 824 genes that have < 5% of 0 TMP values. We then corrected for the GC content bias using an approach that is similar to the one used by EDAseq (Risso et al., 2011). The corrected gene expression values were then quantile normalized.

### 2.2 Bayes factors as evidence for association

For genetic association we used a linear mixed model, fitted by a novel and efficient method that is designed for analyzing multiomics datasets (Guan and Levy, 2024a). The genetic relatedness matrix (twice of kinship matrix) used for the linear mixed model was estimated by Kindred (Guan and Levy, 2024c). The covariates included age, sex, body mass index, and white blood cell composition. This study focuses on genetic association between gene expression and genetic variants in cis, defined as within 1Mb from transcription start site.

We elected to use Bayes factors (Kass and Raftery, 1995) instead of p-values as the evidence of association for the following considerations: 1) Bayes factor has been successfully applied in high profile studies as evidence for genetic association (Wellcome Trust Case Control Consortium, 2007). 2) Bayes factor and p-values have a nice monotone relationship for a fixed allele frequency (Zhou and Guan, 2018; Guan and Levy, 2024b). If an eQTL is significant with Bayes factor, it is likely also significant with p-value, but not vice versa (due to the next point). 3) Bayes factors panelize genetic variants with small allele frequencies to reduce false positives (Guan and Stephens, 2011; Zhou and Guan, 2018). In other words, variants of small allele frequencies may have significant p-values, but unsignificant Bayes factors. This is useful as paternal or maternal allele frequencies can be low by chance even the genotype minor allele frequencies are chosen to be ≥ 0.01. 4) Informally, Bayes factor accounts for multiple testing through prior odds in a manner similar to Bonferroni correction: a smaller prior odds requires larger Bayes factor to maintain the same level of posterior odds for association. 5) Parent-of-origin effects require comparing multiple non-nested modles (e.g., maternal vs. paternal effects). Bayes factors provide a coherent framework for such comparisons, while frequentist approaches lack an equivalent (Stephens and Balding, 2009).

For each gene and its cis-SNP, we computed four Bayes factors: genotype Bayes factor $\left(BF_{g}\right)$, paternal Bayes factor $\left(BF_{1}\right)$, maternal Bayes factor $\left(BF_{0}\right)$, and joint Bayes factors $\left(BF_{j}\right)$. When computing joint Bayes factor, we also computed paternal and maternal effect sizes and attached a p-value to test whether they differ significantly. Examples of test statistics are provided in Table 1. $BF_{g}$, $BF_{0}$ and $BF_{1}$ are one degree of freedom tests, but $BF_{j}$ is a two degree of freedom test. Thus to achieve $BF_{j}>BF_{g}$, the paternal and maternal effect sizes have to differ significantly to compensate for the extra degree of freedom. Examples in Table 1 are chosen because their paternal and maternal effects differ significantly, and we have $BF_{j}\gg BF_{g}$. Another useful observation is that $BF_{1}$ is correlated with $\left|\beta_{1}\right|$, and $BF_{0}$ is correlated with $\left|\beta_{0}\right|$. But $BF_{1}$ and $BF_{0}$ are oblivious to the signs of $\beta_{1}$ and $\beta_{0}$, and signs are informative and interesting in our context.

We used ${\text{log}}_{10}BF=4$ as the significance threshold. If the prior odds for a cis-eQTL is 1 out of 1000, this threshold gives the posterior probability of association (PPA) of 0.91. If the prior odds for a cis-eQTL is 1 out of 100, this threshold gives the PPA of 0.99. 1 − *PPA* is the Bayesian counterpart of the local false discovery rate (c.f. Soloff et al., 2024).

### 2.3 Overview from sentinel eQTL of joint analysis

Table 1 contains 10 genes and test statistics of their sentinel eQTL. These eQTL are chosen because they have most significant column P (see caption for details). A sentinel eQTL for a gene is defined as the eQTL with largest $BF_{j}$ for that gene. A gene with significant eQTL is called eGene. These sentinel eQTL are either $BF_{1}\gg BF_{0}\approx 1$ such as *NDN*, or $BF_{0}\gg BF_{1}\approx 1$ such as *MEG3*, or $BF_{j}\gg BF_{g}\approx 1$ such as *NECAB3*. In Table 1 the sentinel eQTL were ordered according to column P, which measures the significance of differences between paternal and maternal effects. Reassuringly, six out of top ten sentinel eQTL with most significant P are from bona fide imprinted genes (colored in blue) according to http://geneimprint.com .

**Figure 1:**
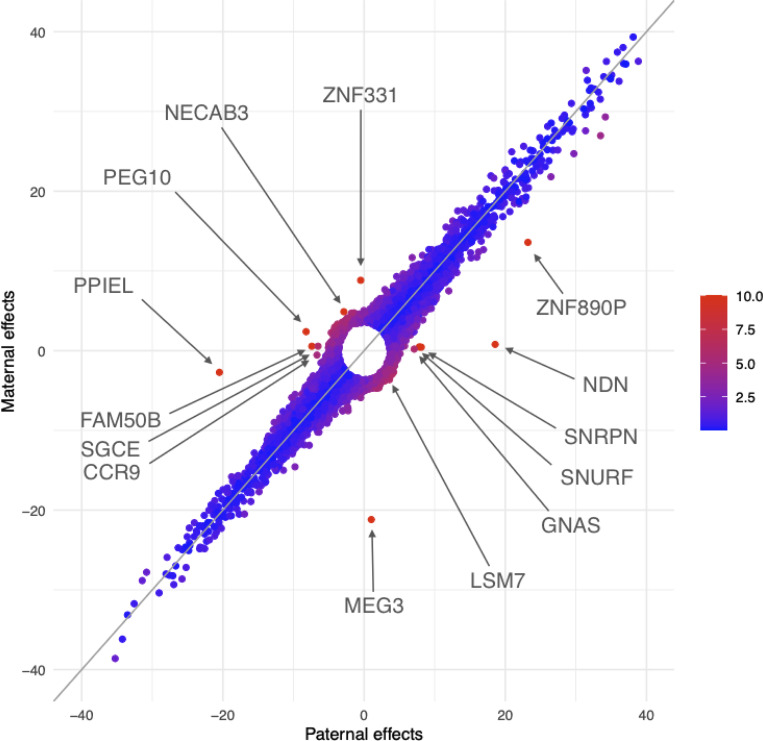
Comparison of paternal effects and maternal effects of sentinel eQTL in joint analysis. Each point is the most prominent eQTL with ${\text{log}}_{10}BF_{j}>3$ of gene. The paternal effect is on x-axis and maternal effect y-axis. The coloring reflects significance of differentials between paternal effects and maternal effects, with red more significant than blue.

Figure 1 plots paternal effect vs maternal effect for all sentinel eQTL whose ${\text{log}}_{10}BF_{j}>3$. The color of dots corresponding to degree of difference between paternal and maternal effects. The marked sentinel eQTL include known imprinted genes such as maternally expressed *ZNF331* and *MEG3*, and paternally expressed *FAM50B, GNAS, SGCE, NDN, SNURF, SNRPN*, and *PEG10*. Imprinted genes are located along the x and y-axes. At least two genes appear to be novel imprinted genes: *PPIEL* and *CCR9*. Gene *ZNF890P* provides an example of paternal and maternal effects being in the same direction, but their sizes differ significantly. Finally, *NECAB3* and *LSM7* are two examples of paternal and maternal effects that are in opposite directions.

This approach based on the sentinel eQTL suggests that 1) the combined effect of paternal and maternal allele can be across all 360 degrees; 2) when paternal and maternal effects are in opposite directions, both effects sizes are modest compared to when they are in the same direction; and 3) there are quite a few sentinel eQTL whose paternal and maternal effects show opposite directions.

### 2.4 *NECAB3* : opposite paternal and maternal effects

**Figure 2:**
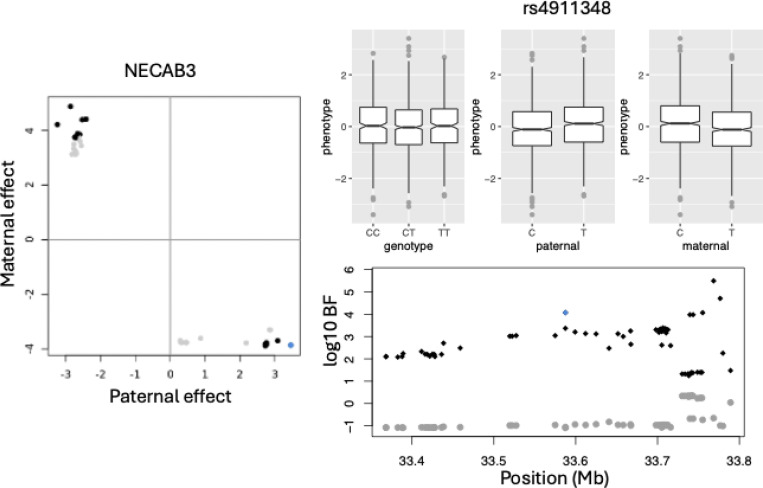
An example of gene that whose paternal and maternal eQTL have opposite effect sizes. On the left is paternal vs maternal effect sizes normalized by their corresponding standard deviation. The effect sizes were estimated by joint analysis. The non-significant eQTL are colored in gray and significant eQTL colored in black. An eQTL marked in blue was chosen to show boxplots with gene expression (phenotype) of NECAB3. The three panels of boxplots are for genotypes, paternal alleles, and maternal alleles. For this SNP rs4911348, the genotypes has no association with phenotype, but both paternal alleles and maternal alleles are associated with the phenotype, and the paternal and maternal effects are in opposite direction. Right panel bottom show Manhattan plot of cis-eQTL of the gene NECAB3. The genotype Bayes factor were colored in gray and joint test Bayes factor were colored in black. SNP rs4911348 was highlighted in blue.

The existence of eGenes whose eQTL show opposite paternal and maternal effects is intriguing. We focused on the example of *NECAB3* to examine further details. Figure 2 left panel shows that all eQTL of *NECAB3* have opposite paternal and maternal effects. Consequently, the joint Bayes factors $BF_{j}$’s are much larger than their corresponding genotype Bayes factors $BF_{g}$’s (right bottom).

We selected as an example SNP rs4911348, which is different from the sentinel eQTL shown in Figure 1, and looked into details of its association with the gene expression of *NECAB3* (phenotype). Three panels of boxplots (right top) show that genotype has no association with the phenotype, and both paternal alleles and maternal alleles are associated with the phenotype, but in opposite directions. Opposite paternal and maternal effects were also observed in Hutterite POE study, where ten associations of opposite effects across nine traits were reported (Mozaffari et al., 2019).

### 2.5 POE eQTL are abundant

Next we looked beyond the sentinel eQTL and examined all significant eQTL. We noted that there are 15, 893 eQTL from 14, 733 SNPs and 1, 824 eGenes that are insignificant for the SNP test, but significant for the paternal, or maternal, or joint test. That is, ${\text{log}}_{10}BF_{g}<4$, but either ${\text{log}}_{10}BF_{1}>4$, or ${\text{log}}_{10}BF_{0}>4$, or ${\text{log}}_{10}BF_{j}>4$. In other words, these eQTL cannot be detected without analyzing POE, which implies that POE can contribute to recover missing heritability.

With reference Figure 1, we proposed criteria to define subsets of eQTL. The first subset eQTL locate along the x-axis, mimicking sentinel eQTL of *NDN* and *FAM50B*; the second subset locate along the y-axis, mimicking sentinel eQTL of *ZNF331* and *MEG3*; the third subset locate along the secondary diagonal, mimicking sentinel eQTL of *NECAB3* and *LSM7*; and the fourth subset follows (blues dots) along the diagonal line. Note Figure 1 only involves $BF_{j}$, our criteria to define gene sets also takes into account of $BF_{1}$, $BF_{0}$, and $BF_{g}$ (details in Methods).

The first set of eQTL have significant paternal effects but insignificant maternal effects. We identified 15, 576 such *paternal eQTL* (set $S_{P}$) from 14, 372 SNPs associated with 1, 188 eGenes. The second set of eQTL have significant maternal effects but insignificant paternal effects. We identified 14, 783 such *maternal eQTL* (set $S_{M}$) from 13, 293 SNPs associated with 1, 209 eGenes. We refer to these paternal and maternal eQTL as *imprinting eQTL*. The third set of eQTL have opposite paternal and maternal effects, such that joint Bayes factors are much larger than genotype Bayes factor $BF_{j}\gg BF_{g}$. We identified 688 such *opposing eQTL* (set $S_{O}$) from 485 SNPs that are associated with 180 eGenes. Imprinting eQTL and opposing eQTL are referred to as *POE eQTL*.

The fourth set of eQTL require that both paternal and maternal alleles alone are associated with an expression phenotype, and the two effects are in the same direction. For these eQTL $BF_{g}>BF_{j}$ because similar effect sizes favor one degree of freedom test. We identified 884, 119 such *genotype eQTL* from 577, 701 SNPs and 4940 eGenes. The percent of eGenes with genotype eQTL is on par, but smaller than GTEx study (Consortium et al., 2020), presumably because ${\text{log}}_{10}BF_{g}>4$ is a more stringent threshold for eQTL analysis.

### 2.6 Paternal eQTL are enriched with GWAS hits

Using ANNOVAR (Wang et al., 2010), we annotated all four sets of eQTL (Table 2). We made the following observations: 1) The median distance to transcription start site (TSS) for opposing eQTL is 673.2Kb, much larger than other types of eQTL, and majority of opposing eQTL $S_{O}$ are located in introns of nearby genes. 2) The percent of GWAS hits for SNPs in $S_{O}$ is 0.022, significantly lower than that of SNPs in genotype eQTL $S_{G}$’s 0.054 (test for proportion *P* = 0.001). This is reasonable as GWAS mainly use genotype test and are agnostic to opposing eQTL. 3) The precent of GWAS hits for SNPs in paternal eQTL $S_{P}$ is higher than that of SNPs in $S_{G}$ (test for proportion *P* = 9 × 10^−11^), and the percent of GWAS hits among SNPs in maternal eQTL $S_{M}$ has no significant difference to that of SNPs in $S_{G}$ (test for proportion *P* = 0.051).

Number of eQTL per SNP (r) measures degree of pleiotropy of a set of SNPs. SNPs in $S_{P}$ and SNPs in $S_{M}$ have similar pleiotropy r≈1.1, SNPs in $S_{G}$ have the largest r=1.53, and SNPs in $S_{O}$ display r=1.41. In other words, SNPs are more exclusive for imprinting eQTL, less exclusive for genotype eQTL, and somewhere in between for opposing eQTL. Table 2 (bottom) compares how the degree of pleiotropy affects their annotation for SNPs in $S_{G}$, where set $P_{k}$ contains SNPs in $S_{G}$ that are eQTL of at least k eGenes. The most interesting observation is that the percent of GWAS hits increases with rising SNP pleiotropy. This feature is only observed in genotype eQTL.

### 2.7 POE eGenes are abundant

There are 1, 188 eGenes harboring paternal eQTL and 1, 209 eGenes harboring maternal eQTL. Their union contains 2, 139 eGenes harbor imprinting eQTL, and their intersection contains 258 eGenes harboring both paternal and maternal eQTL. There are 4, 940 eGenes harboring genotype eQTL, and 1, 867 eGenes harboring both genotype eQTL and imprinting eQTL. Supplementary Figure S1 showed an example of an eGene harboring both maternal and paternal eQTL: *GZMH*, and an example of an eGene harboring both genotype eQTL and imprinting eQTL: *DSE*. The most prominent feature is that paternal eQTL and maternal eQTL cluster in separate genomic regions, and genotype eQTL and imprinting eQTL also cluster in separate genomic region.

### 2.8 eGenes that harbor exclusively imprinting eQTL

There are 129 eGenes (set $G_{1}$) that harbor exclusively paternal eQTL, and 139 eGenes (set $G_{0}$) that harbor exclusively maternal eQTL. Naturally, $G_{1}$ contain many bona fide imprinted genes with paternal expression such as *FAM50B, NDN, SGCE, SNRPN, SNURF* and *PEG10*. Remaining genes in $G_{1}$ are candidates for imprinted genes with paternal expression, for example *CCR9* and *NCOA2*. $G_{0}$ contain bona fide imprinted genes with maternal expression such as *GRB10, ZNF331* and *MEG3*. The remaining in $G_{0}$ are candidates for imprinted genes with maternal expression, for example *COA8* and *ZNF888*.

The Supplementary Material provides a full list of the candidate imprinted genes from the eQTL analysis. Note however, that not all imprinted genes have eQTL, therefore this list is incomplete. Moreover, a gene on the list is considered behaving like an imprinted gene, but may not be a bona fide imprinted gene. For example, a gene that has no cis-eQTL on its own, may “acquires” a cis-eQTL through gene-gene interaction with a putative imprinted gene such that the cis-eQTL is in fact its trans-eQTL.

We used $G_{1}$ and $G_{0}$ as proxies for paternal and maternal imprinted genes, and used cytoBands these genes occupies as proxies for imprinted regions to examine to what extend paternal and maternal eQTL locate in the same cytoBands as these proxy imprinted genes. There are 811 autosome cytoBands in humans, 765 of them larger than 1 Mb, and the median size is 3.2 Mb (Cheung et al., 2001). The 129 proxy paternal imprinted genes occupy 89 cytoBands (set $C_{P_{I}}$), while 15, 576 paternal eQTL occupy 445 cytoBands (set $C_{P_{e}}$), among them 9, 947 (or 64%) were not in the cytoBands set $C_{P_{I}}$. On the other hand, the 139 proxy maternal imprinted genes occupy 96 cytoBands (set $C_{M_{I}}$), and 14, 783 maternal eQTL occupy 465 cytoBands (set $C_{M_{e}}$), among them 7, 987 (or 54%) were not in the cytoBand set $C_{M_{I}}$. Therefore, a majority of imprinting eQTL are not in the vicinity of the proxy imprinted regions. Interestingly, $C_{P_{I}}$ and $C_{M_{I}}$ share only 14 cytoBands, 8% of their union of 171 cytoBands. As a comparison, $C_{P_{e}}$ and $C_{M_{e}}$ share 349 cytoBands, 62% of their union of 561 cytoBands.

### 2.9 eGenes that harbor exclusively opposing eQTL

There are 99 eGenes (set $G_{2}$) that harbor only opposing eQTL (Supplementary). Among them 58 encode phosphoproteins (Supplementary), a significant enrichment (FDR = 5.5 × 10^−4^) according to DAVID, a web server for functional enrichment analysis (Sherman et al., 2022). A phosphoprotein is a protein that is posttranslationally modified by the attachment of either a single phosphate group, or a complex molecule such as 5’-phospho-DNA, through a phosphate group. Because phosphorylation often serves as an on/off switch, targeting phosphoproteins or the enzymes regulating them can restore normal cell signaling in diseases. In addition, drugs can modulate the functions of these proteins, either inhibiting or enhancing their activities. This makes phosphoprotein attractive drug targets. Indeed, according to a therapeutic target database (Zhou et al., 2024), among 58 phosphoproteins genes that harbor exclusively opposing eQTL, there are three successful drug targets *ITPR1, ITPR2*, and *REL*, and three additional clinical trial targets *CD46, NOTCH3*, and *XPO1*.

Other notable phosphoproteins genes include *CDKN2A*, *PHIP*, and *LEP*, among others. Where *CDKN2A* produces two major proteins p16(*INK4*), which is a cyclin-depnednet kinase inhibitor, and p14(*ARF*), which binds the p53-stabilizing protein MDM2 (Serrano et al., 2000); *PHIP* is associated with Chung-Jansen syndrome, featuring behavioral problems, intellectual disability, obesity, and dysmorphia (Webster et al., 2016; Jansen et al., 2018); and *LEP* encodes leptin, a protein that plays a critical role in the regulation of body weight. Leptin is secreted by white adipocytes, and it binds to the leptin receptor in the brain, which in turn inhibits appetite and promotes energy expenditure (Maffei et al., 1995; Weigle et al., 1997).

### 2.10 Paternal eGenes are enriched with drug targets

We used a therapeutic target database (Zhou et al., 2024) to examine the enrichment with drug targets of the four sets of eGenes, grouped by their eQTL. Table 3 contains the enrichment of the four gene sets in either successful targets, clinical trial targets, or combined targets. The significant enrichment that (following Bonferroni correction of 12 tests) is highlighted. Both paternal eGenes and genotype eGenes are enriched in clinical trial targets and combined targets, but paternal eGenes are significantly more enriched than genotype eGenes (one-sided test for proportion *P* = 0.035). The enrichment of drug targets for paternal eGenes echoes the enrichment of GWAS hits for paternal eQTL (Table 2). This asymmetry between paternal and maternal eGenes perhaps finds its root in the conflicting

interests of two sets of genes in relation to transfer of nutrients from the mother to her offspring (Moore and Haig, 1991). It is known that paternal alleles may have distinct evolutionary and functional roles compared to maternal alleles. Maze serves as an extreme example, where gene expression of the hybrid is regulated exclusively by the paternally transmitted alleles (Swanson-Wagner et al., 2009).

## 3 Discussion

Our study provides a comprehensive analysis of POE eQTL in human gene expression using data from the Framingham Heart Study. The extensive presence and complexity of POE uncovered in this study have significant implications for our understanding of genetic regulation and its role in complex traits and diseases. To illustrate the implications in the context of existing research, we revisit two previous findings in light of our new results, and advise caution on conducting Mendelian randomization using gene expression as the exposure.

### 3.1 T2D and maternal allele of rs2237892

SNP rs2237892 in the last intron of *KCNQ1* was found in association with type 2 diabetes (T2D) in a Japanese cohort (Yasuda et al., 2008), and the association was later confirmed in a Chinese cohort (Liu et al., 2009). Three other SNPs (rs2283228, rs2237895, and rs2237897) in the same last intron of *KCNQ1* were found in association with T2D in another Japanese cohort (Unoki et al., 2008). Both rs2237895 and rs2237897 were replicated in a Singaporean cohort, a Danish cohort (Unoki et al., 2008), and a Chinese cohort (Liu et al., 2009). In these studies, *KCNQ1* was identified as T2D candidate gene.

Based on these results from the genotype test, Kong et al. (2009) demonstrated that SNP rs2237892 was maternally associated with T2D in an Iceland cohort. This maternal association was replicated with a larger odds ratio in a Pima Amerindian cohort (Hanson et al., 2013). According to data from GTEx, none of these SNPs are eQTL of *KCNQ1* and *CDKN1C* in any tissue, consistent with the fact that these are imprinted genes and GTEx study is oblivious to parent of origin. Our eQTL analysis shows that SNPs rs2237892, rs2283228, and rs2237897 are maternal eQTL of *CDKN1C*, a down-stream neighbor of *KCNQ1*, and none of these SNPs are eQTL of *KCNQ1*.

We therefore suggest that *CDKN1C*, instead of *KCNQ1*, is a T2D candidate gene, for the following reasons: 1) These GWAS SNPs connect T2D and *CDKN1C* quantitatively, but connect T2D and *KCNQ1* only geographically; 2) A study suggests that *CDKN1C* mutations may represent a novel monogenic form of diabetes (Kerns et al., 2014); 3) A boy carrying a frameshift mutation in *CDKN1C* was diabetic from week 29 (Berland et al., 2022); and 4) Targeted demethylation at the CDKN1C/p57 locus induces human β cell replication, while the loss of insulin-secreting β cell is characteristic among T1D and T2D (Ou et al., 2019).

### 3.2 Polar overdominance and rs1802710

Overdominance is a pattern of inheritance such that both homozygous AA and BB have the same baseline trait value, while heterozygous AB has a higher trait value. Polar overdominance (Cockett et al., 1996) introduces asymmetry into overdominance to separates AB into two groups according to parent-of-origin of A (or B), such that AB with paternal A (or B) has a high trait value, while AB with maternal A (or B) has the baseline trait value.

Wermter et al. (2008) studied trios of extremely obese offspring and identified rs1802710 in exon 5 of *DLK1*, homologous to the ovine callipyge locus (Cockett et al., 1996), whose allelic transmission pattern was consistent with polar overdominance: frequent transmission of the paternal C allele to obese children, but the relative risk for carriers of the homozygous CC genotype was not increased compared to the reference TT genotype.

Our eQTL results show that *DLK1* has no significant eQTL, but the maternal C allele of rs1802710 reduces expression of *MEG3*, 53Kb downstream of *DLK1*. Both *MEG3* and *DLK1* are imprinted with *MEG3* maternally expressed and *DLK1* paternally expressed. The expression of *MEG3* was shown to be significantly higher in the obese group (Daneshmoghadam et al., 2021). Therefore the maternal C allele is associated with reduced risks of obesity, which balances out the increased risk conferred by paternal C allele, thus offering an explanation for the baseline risk of homozygous CC samples in (Wermter et al., 2008). Since rs1802710 is not an eQTL of any gene in any tissue according to GTEx data, our study is critical to link rs1802710 with *MEG3*, albeit not in the adipose tissue.

### 3.3 Mendelian randomization

Mendelian randomization (MR) refers to the random allocation of alleles at the time of gamete formation. Observational epidemiology studies use MR to infer the causal effect of an exposure on an phenotype (Smith and Ebrahim, 2003), as if the random allocation of alleles is comparable to a randomized clinical trial. An important assumption for MR, among many other important assumptions, is that the phenotype conferred by a specific genetic variant is homogeneous in the population, and exchangeable between paternally and maternally inherited alleles. POE is a direct violation of this assumption (Bochud et al., 2008).

There is a growing interest in using gene expression as an exposure to perform MR across various traits (Porcu et al., 2019; van der Graaf et al., 2020). If an exposure has eQTL with POE, ignoring the parent-of-origin will bias prediction of a subset of samples. If the bias is severe it will change the ranking of the exposure. Consequently, it either compromises the power of the MR or leads to a false conclusion of a causal relationship between the exposure and the phenotype.

Our study demonstrated the abundance of POE in gene expressions, and therefore advised caution when conducting MR using gene expression as the exposure. Our suggestion is checking the list of POE SNPs and eGenes we provided in the Supplementary Material and excluding those that show POE towards the exposure. The same suggestion is also applicable to computing polygenic risk scores, and imputing gene expression such as PrediXcan (Gamazon et al., 2015) and a Bayesian method motivated by it (Qi et al., 2018).

### 3.4 Limitations of the Study

Despite the significant findings, our study has several limitations. First, the analysis is restricted to whole blood gene expression, which may not capture POE in other tissues. Gene expression and POE can be tissue-specific, and further studies are needed to investigate POE across different tissues to gain a comprehensive understanding of its impact.

Second, the study relies on the availability of trios with both whole genome sequencing and RNA sequencing data, which limits the sample size. Although the Framingham Heart Study provides a relatively large number of trios, increasing the sample size and including more diverse populations would enhance the generalizability of our findings.

Finally, the functional implications of the identified POE eQTL require further validation through experimental studies. While our study provides a comprehensive catalog of POE eQTL, understanding their biological significance and mechanisms of action will require detailed experimental investigations.

### 3.5 Conclusion

Our study highlights the abundance and the complexity of POE in gene expression, providing a valuable resource that complements existing databases such as GTEx. These findings have significant implications for genetic association studies, the interpretation of complext traits, and the development of targeted therapies. By expanding the understanding of POE, this study contributes to a deeper comprehension of genetic regulation and its impact on human health.

## 4 Methods

### 4.1 Genotype data

We first used pedigree and availability of RNAseq data to narrow the samples down to 2955 individuals and identified all bi-allelic SNPs following a TOPMed protocol (Taliun et al., 2021). We then used Kindred to infer pairwise kinship for each chromosome, and computed mean and standard deviation (SD) of the kinship. A sample pair that is parent-offspring can be distinguished from full sibs by the SD (Supplementary Figure S2). Trios are consisted of sample A, B, and C such that ϕ(A,B)≈0 and ϕ(A,C)≈ϕ(B,C)≈0.25 where ϕ(⋅,⋅) is the kinship. In the end, we identified 1477 trios that have whole genome sequencing data and whose children have RNAseq data. In total, we had 7, 752, 281 autosomal bi-allelic SNPs with minor allele frequency > 0.01 (not corrected for relatedness). Across all 1477 trios, the Mendelian error is negligible 4.76 per 100, 000 SNPs per trio. To handle Mendelian errors, we assumed that the child was correctly genotyped, and errors occurred in the parents, but if the child is heterozygous, we assumed this to be triple heterozygous SNP. On average 1477 trios had 65 triple heterozygous genotypes per SNP. The triple heterozygous SNPs cannot be phased by Mendelian inheritance, but can be phased based on a linkage disequilibrium model (below).

### 4.2 Inference of parent of origin

For each trio, we first phased all markers that are not triple heterozygous using rules of Mendelian inheritance, where for the child in each trio we obtained paternal and maternal haplotypes with triple heterozygous markers whose phase were unresolved. We marked those triple heterozygous markers as missing. For markers that showed Mendelian incompatibility, we assumed that the child’s genotype was correct and marked it as missing if it was heterozygous. Using a hidden Markov model that was designed to model haplotype variation (Guan, 2014), we imputed missing markers in each haplotypes to obtain dosage estimates (between 0 and 1). Then for each marker that was marked as missing, we compared imputed dosages between paternal and maternal haplotypes, and assigned the large dosage as 1 and small dosage as 0. We thus obtained paternal and maternal haplotypes with no missing data. We simulated trios using haplotypes from the 1000 Genomes project (Auton et al., 2015), and investigated the accuracy of the phasing by the mask and imputation approach described above. The phasing was highly accurate, the error rate was less than 1 out of 200 triple heterozygous SNPs (Supplementary Table S1).

### 4.3 RNAseq data

The RNAseq data of 1477 children was obtained in three batches with 1381 in batch 1; 14 in batch 2, and 82 in batch 3. Our primary goal was to correct for GC content bias. To this end, we first removed genes whose proportion of 0 TPM values was greater than 5%, which retained 16, 969 genes out of total 58103, a majority of which are coding genes. Among those, 16, 824 genes have GC content. To correct for GC content bias, we took an approach used by EDAseq to regress out the GC content from log(1+TMP), but instead of using local linear regression as documented in EDAseq, we used local quadratic regression implemented in loess in R (Supplementary Figure S3). Specifically, let y=log(1+TMP) be a gene expression and g be the GC content, we fit ly=loess(y,g) and compute $\hat{y}=median\text{(}y\text{)+}ly\text{\$}residuals$. We then quantile normalized $\hat{y}$ by $qqnorm(\hat{y},plot.it=F)\$y$ (in R) separately for each gene.

### 4.4 eQTL analysis

We used IDUL to perform eQTL analysis. IDUL fits linear mixed models to achieve exact optimal. It was specifically developed to analyze multi-omics data to achieve high efficiency by reusing the intermediate computations (Guan and Levy, 2024a). Previous analysis showed that for the Framingham Heart Study participants, the genomic inflation was well controlled using a linear mixed model with kinship matrix computed by Kindred (Guan and Levy, 2024c). In this study, we also controlled age, sex, BMI, and white blood cell compositions in peripheral blood. We now briefly describe how Bayes factors were computed. Consider a model

(1) $$\text{y}=\text{Wa}+\text{x}\beta +\text{Zu}+\text{e}\;\text{u}∼\text{MV}{\text{N}}_{n}\left(0,\tau^{-1}\eta \text{K}\right)\;\text{e}∼\text{MV}{\text{N}}_{n}\left(0,\tau^{-1}{\text{I}}_{n}\right)$$

where W contains conventional covariates such as age and sex, including a column of 1, x contains genetic variant(s) to be tested for association, u is the random effect with Z as its loading matrix and twice of kinship K as its covariance (both Z and K are known), $\text{MV}{\text{N}}_{n}$ denotes an n-dimensional multivariate normal distribution, ${\text{I}}_{n}$ is n-dimensional identity matrix. Denote X=(W,x) and b=(a,β), then Xb is the fixed effect, and we assume X has a full rank c. In genetic association studies, the random effect Zu is a nuisance term that absorbs part of the phenotype y that is attributable to population stratification and relatedness. The maximum likelihood estimate (MLE) of η can be efficiently obtained (Guan and Levy, 2024a), plug $\hat{\eta }$ back into (1), and specify the following conjugate prior

(2) $$\text{a}∼N\left(0,\tau^{-1}V_{\text{a}}\right)\;\beta ∼N\left(0,\tau^{-1}\sigma^{2}\right)\;\tau ∼\text{Γ}\left(\kappa_{1}/2,\kappa_{2}/2\right)$$

and let $V_{\text{a}}\to \infty$, $\kappa_{1}\to 0$ and $\kappa_{2}\to 0$, Bayes factor can be evaluated efficiently in a closed form. In this study we used σ=0.5, following prior work of Bayes factors for linear models (Servin and Stephens, 2007). Complete details on computing Bayes factors for linear mixed model can be found in (Guan and Levy, 2024b).

### 4.5 P-value for difference between paternal maternal effects

For joint analysis, we have

(3) $$\text{y}=\text{Wa}+{\text{x}}_{1}\beta_{1}+{\text{x}}_{2}\beta_{2}+\text{Zu}+\text{e}$$

with the prior for $\beta_{1}$ and $\beta_{2}$ as

(4) $$\beta_{1}∼N\left(0,\tau^{-1}\sigma^{2}\right)\;\beta_{2}∼N\left(0,\tau^{-1}\sigma^{2}\right)$$

The Bayes factor can be similarly computed in a closed form. We obtain the posterior estimates of ${\hat{\beta }}_{1}$ and ${\hat{\beta }}_{2}$, their variances $s_{1}^{2}$ and $s_{2}^{2}$ and covariance $s_{12}$. To test null hypothesis ${\hat{\beta }}_{1}={\hat{\beta }}_{2}$, we compute test statistics $t=\frac{{\left({\hat{\beta }}_{1}-{\hat{\beta }}_{2}\right)}^{2}}{s_{1}^{2}+s_{2}^{2}-2s_{12}}$. Since under the null t following $\chi^{2}$ distribution with 1 degree of freedom, we can compute a p-value.

### 4.6 Threshold for eQTL sets and gene sets

For paternal eQTL set $S_{P}$, we required ${\text{log}}_{10}BF_{1}>4$ and ${\text{log}}_{10}BF_{0}<\theta$; For maternal eQTL set $S_{M}$, we required ${\text{log}}_{10}BF_{0}>4$ and ${\text{log}}_{10}BF_{1}<\theta$; For opposing eQTL set $S_{O}$, we required ${\text{log}}_{10}BF_{j}-{\text{log}}_{10}BF_{g}>4$ and ${\text{log}}_{10}BF_{1}>\theta$ and ${\text{log}}_{10}BF_{0}>\theta$, and $\beta_{1}*\beta_{0}<0$; For genotype eQTL set $S_{G}$, we required ${\text{log}}_{10}BF_{g}>4$ and ${\text{log}}_{10}BF_{1}>\theta$ and ${\text{log}}_{10}BF_{0}>\theta$, and $\beta_{1}*\beta_{0}>0$. It’s easy to see for a particular type of eQTL, different θ produced nested eQTL sets. We tried θ=0, ${\text{log}}_{10}\;2$, and ${\text{log}}_{10}\;3$ to obtain eQTL sets (Supplementary Table S2), from which we obtained gene sets harboring those eQTL $G_{P}$, $G_{M}$, $G_{A}$ and $G_{G}$, from which we obtained genes harboring exclusively paternal eQTL $G_{1}=G_{P}/\left(G_{M}\cup G_{A}\cup G_{G}\right)$, genes harboring exclusive maternal eQTL $G_{0}=G_{M}/\left(G_{P}\cup G_{A}\right.\left.\cup G_{G}\right)$, and genes harboring exclusively opposing eQTL $G_{0}=G_{A}/\left(G_{P}\cup G_{M}\cup G_{G}\right)$. We compared known imprinted genes with paternal expression with $G_{1}$, and known imprinted genes with maternal expression with $G_{0}$. $\theta ={\text{log}}_{10}2$ produced minimum $G_{1}$ and $G_{0}$ that contain known imprinted genes.

### 4.7 Availability of data and materials

This study analyzed existing datasets from public domain and simulated datasets. Data from the 1000 Genomes project can be found at https://www.internationalgenome.org, and datasets from Framingham Heart Study can be obtained via dbGaP. The pipeline and software used to conduct analysis is available at https://github.com/haplotype/poe. The results (POE eQTLs) generated from current study will be made available to general public once the manuscript is in print.

## Supplementary Material

Supplement 1

## Figures and Tables

**Table 1:** Example of eQTL statistics. $lgBF_{x}$ columns are ${\text{log}}_{10}BF_{x}$. Column $\beta_{j}$’s are effect estimates in joint analysis used to compute $BF_{j}$. P column contains ${-\text{log}}_{10}$ p-value with the null hypothesis $\beta_{0}=\beta_{1}$ and alternative hypothesis $\beta_{0}\neq \beta_{1}$. Examples chosen here have most significant P among sentinel eQTL. Transcript start site (TSS) is in Mb, and the coordinate is from HG38. Bona fide imprinted genes are colored in blue.

Gene	TSS	SNP	$lgBF_{g}$	$lgBF_{1}$	$lgBF_{0}$	$lgBF_{j}$	$\beta_{1}$	$\beta_{0}$	P

MEG3	100.78	rs12881545	35.7	−0.7	86.3	85.7	1.0	−21.2	54.0
PPIEL	39.53	rs79473113	47.6	84.6	0.5	85.8	−20.5	−2.7	38.3
NDN	23.69	rs3743340	36.5	68.5	−0.2	67.7	18.6	0.8	33.2
PEG10	94.66	rs7801134	2.6	13.3	−0.1	13.6	−8.2	2.4	12.6
ZNF331	53.52	rs1284523	6.3	−0.9	15.9	14.9	−0.5	8.8	10.3
ZNF890P	5.12	rs112844843	125.8	94.7	31.1	133.9	23.2	13.6	10.1
FAM50B	3.85	rs111515624	3.5	11.5	−0.5	10.9	−7.4	0.6	8.6
NECAB3	33.66	rs2626556	−0.7	1.2	4.6	5.5	−2.9	4.9	7.8
SNURF	24.95	rs4906936	5.1	12.1	−0.9	11.2	7.7	−0.1	7.6
LSM7	2.32	rs393651	−1.0	3.5	1.6	4.9	4.4	−3.3	7.5

**Table 2:** Top tabular: Annotation of eQTL. $S_{O}$ is a set of opposing eQTL, $S_{P}$ is a set of paternal eQTL, $S_{M}$ is a set of maternal eQTL, and $S_{G}$ is a set of genotype eQTL. The column eQTL contain counts of eQTL in each set, and column SNP contains counts of distinct SNPs of eQTL in the set. The column *eGene* is the number of genes associated with eQTL in the set. The column of Len is the median length in Kb of the eGenes. (The pattern is the same with the mean length.) The column $D_{TSS}$ contains median distance in Kb to transcription start site. The column GWAS contains percent of GWAS hits among SNPs, with GWAS p-value threshold of 5 × 10^−8^. Bottom tabular: A SNP set $P_{k}$ contain SNPs in $S_{G}$ that are eQTL of at least k eGenes. (Note $P_{1}=S_{G}$.) Percent of GWAS hits for SNP set $P_{k}$ increases with k.

Set	eQTL	eGene	Len	*D*_*TSS*_	Genic	Intron	Exon	ncRNA	SNP	GWAS
$S_{O}$	688	180	28.1	**673.2**	0.260	**0.663**	0.006	0.026	485	**0.023**
$S_{P}$	15,576	1188	23.3	122.9	0.362	0.490	0.016	0.080	14,372	**0.066**
$S_{M}$	14,783	1209	23.8	122.5	0.365	0.487	0.016	0.076	13,293	0.050
$S_{G}$	884,119	4940	24.6	84.1	0.383	0.449	0.017	0.091	577,701	0.054

$P_{1}$	884,119	4940	24.6	84.1	0.383	0.449	0.017	0.091	577,701	0.054
$P_{2}$	471,354	2855	17.7	96.8	0.400	0.403	0.019	0.112	164,936	0.071
$P_{3}$	269,546	1536	13.8	114.4	0.440	0.349	0.020	0.129	64,032	0.088
$P_{4}$	172,871	858	11.0	134.2	0.488	0.295	0.017	0.143	31,807	0.097
$P_{5}$	126,107	510	8.6	143.8	0.508	0.284	0.016	0.140	20,116	0.110
$P_{6}$	94,367	383	7.7	146.7	0.520	0.284	0.015	0.135	13,768	0.117
$P_{7}$	46,925	272	6.1	131.8	0.593	0.234	0.015	0.106	5,861	0.133
$P_{8}$	26,695	178	3.7	116.6	0.619	0.221	0.010	0.099	2,971	0.144
$P_{9}$	12,783	124	2.3	121.8	0.483	0.355	0.011	0.111	1,232	0.198

**Table 3:** Enrichment of drug target genes. $G_{O}$ contains eQTL with both paternal and maternal effects but they are in opposite direction. $G_{P}$ contains eQTL of paternal effects. $G_{M}$ contains eQTL of maternal effects. $G_{G}$ contain eQTL that have both paternal and maternal effects and they are in the same direction. Column nGene are sizes of gene sets, Column nTarget are counts of successful drug targets. The last column contains one-sided test-of-proportion p-values. The highlighted p-values are significant after Bonferroni correction of 12 tests.

	Set	eGenes	nTarget	$\hat{f}$	$f_{0}$	$P\left(\hat{f}>f_{o}\right)$

Successful target	$G_{O}$	180	7	0.0389	0.0220	0.098
	$G_{P}$	1188	38	0.0320	0.0220	0.012
	$G_{M}$	1209	23	0.0190	0.0220	0.728
	$G_{G}$	4940	124	0.0251	0.0220	0.075

Clinical trial target	$G_{O}$	180	7	0.0389	0.0421	0.512
	$G_{P}$	1188	69	**0.0581**	0.0421	**0.004**
	$G_{M}$	1209	67	0.0554	0.0421	0.013
	$G_{G}$	4940	254	**0.0514**	0.0421	**0.0006**

Combined target	$G_{O}$	180	14	0.0778	0.0641	0.275
	$G_{P}$	1188	107	**0.0901**	0.0641	**0.0002**
	$G_{M}$	1209	90	0.0744	0.0641	0.079
	$G_{G}$	4940	374	**0.0757**	0.0641	**0.0005**
