# Supplementary material for: Abundant Parent-of-origin Effect eQTL: The Framingham Heart Study
Source: bioRxiv. 2025 Jun 4:2024.06.05.597677. Preprint. [Version 2] doi: 10.1101/2024.06.05.597677 (PMC12157689; doi:10.1101/2024.06.05.597677)
Supplement: Supplement 1 [file media-1.pdf]

# Abundant Parent-of-origin Effect eQTL: The Framingham Heart Study (Supplementary)

Yongtao Guan, Tianxiao Huan, and Daniel Levy
National Heart, Lung, and Blood Institute

June 4, 2025

## 1 Simulation study of phasing trios

There are 503 European (EUR), 504 East Asian, and 661 African (AFR) phased samples in 1000 Genomes project. Our simulation was conducted separately for each population. In each population, we randomly selected two samples without replacement and assign the first as father and the second as mother, until exhausted all samples or had only one sample left. Using Chromosome 22, we selected one haplotype from the father and one from the mother to simulate the child. We didn't simulate genotyping error, as in real data the Mendelian error is negligibly small. We then ran the process of phasing by rule of Mendelian inheritance for each trio, masked triple heterozygous SNPs for each child, jointly fit an LD model using all chidren's haplotypes to impute masked (heterozygous) SNPs, and finally based on imputed allele dosage to assign alleles (reference or alternative) to maternal and paternal haplotypes. We tallied all counts and put in Table S1, and results show our approach of phasing is highly accurate.

| | EUR | EAS | AFR |
|---|---|---|---|
| $N_I$ | 251 | 252 | 330 |
| $N_S$ | 30,501 | 30,551 | 30,551 |
| $N_{3H}$ | 882,076 | 837,090 | 1,179,930 |
| $N_C$ | 878,711 | 833,192 | 1,175,340 |
| $R$ | 0.996 | 0.995 | 0.996 |

Table S1: Statistics and accuracy of phasing triple heterozygous sites via mask and imputation. $N_i$: number of simulated samples. $N_s$ number of biallelic SNPs used in simulation. $N_{3H}$: number of triple heterozygous SNPs across $N_i$ samples. $N_T$: number of triple heterozygous SNPs that being correctly phased. $R$: ratio of the correctly phased triple heterozygous SNPs. EUR: European samples. EAS: East Asian samples. AFR: African samples.

# 2 Examples of eGenes harboring different set of eQTL

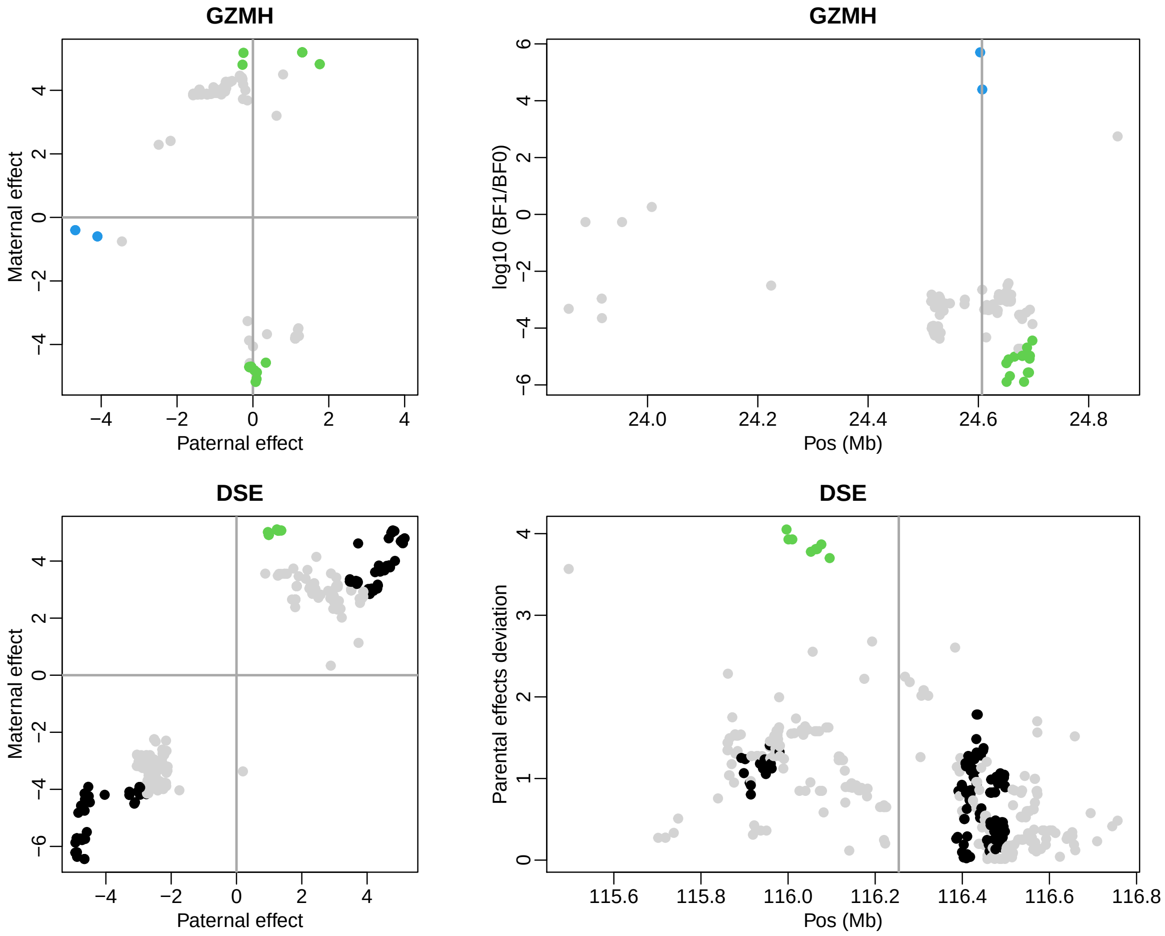


Figure S1: Examples of eGenes harboring different sets of eQTL: *GZMH* and *DSE*. Each gene has two plots: a square plot showing normalized paternal effect (x-axis) vs normalized maternal effect (y-axis), and a rectangle plot showing test statistics along chromosome position. In each plot, gray dots are insignificant eQTL, black dots are significant genotype eQTL, blue dots are significant paternal eQTL, and green dots are significant maternal eQTL. The vertical line in the right panels mark the transcription start site.

## 3 Kinship estimates

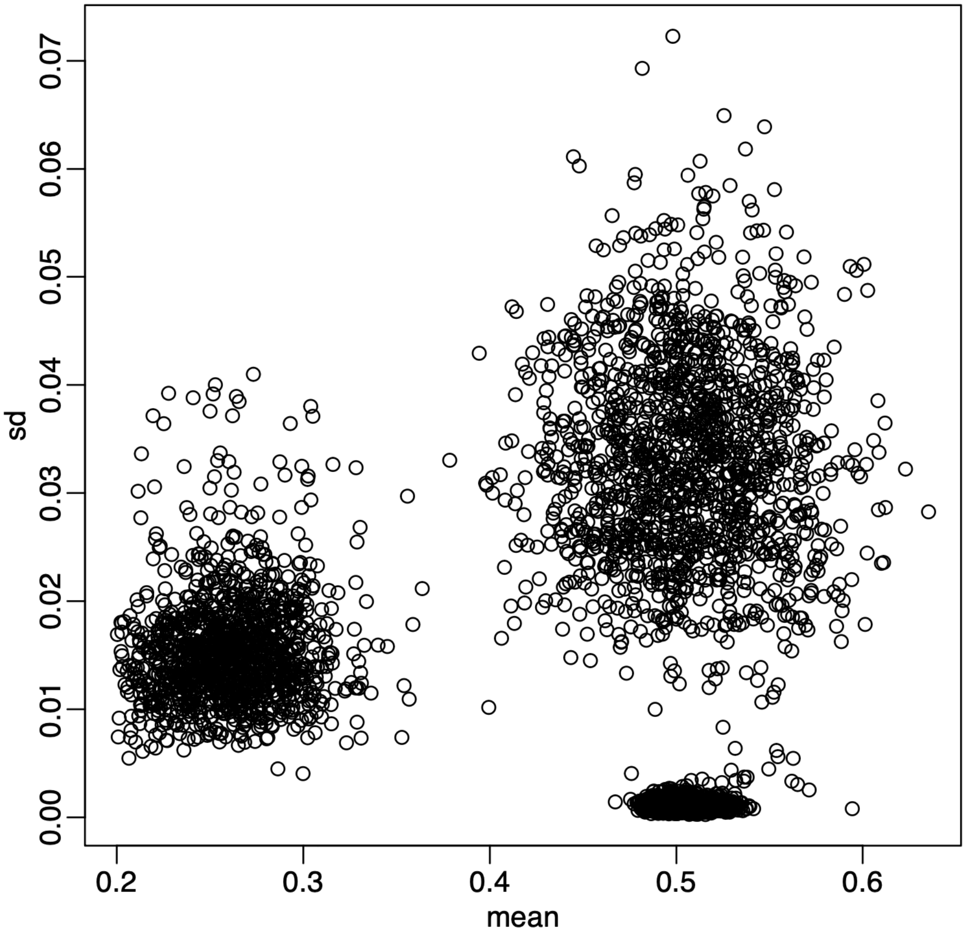


Figure S2: Kinship and sd. The x-axis is twice of kinship averaged over 22 estimates, one for each autosome. The y-axis is standard deviation of those 22 estimates. Plot only show relevant portion of the kinship.

# 4 GC content bias correction

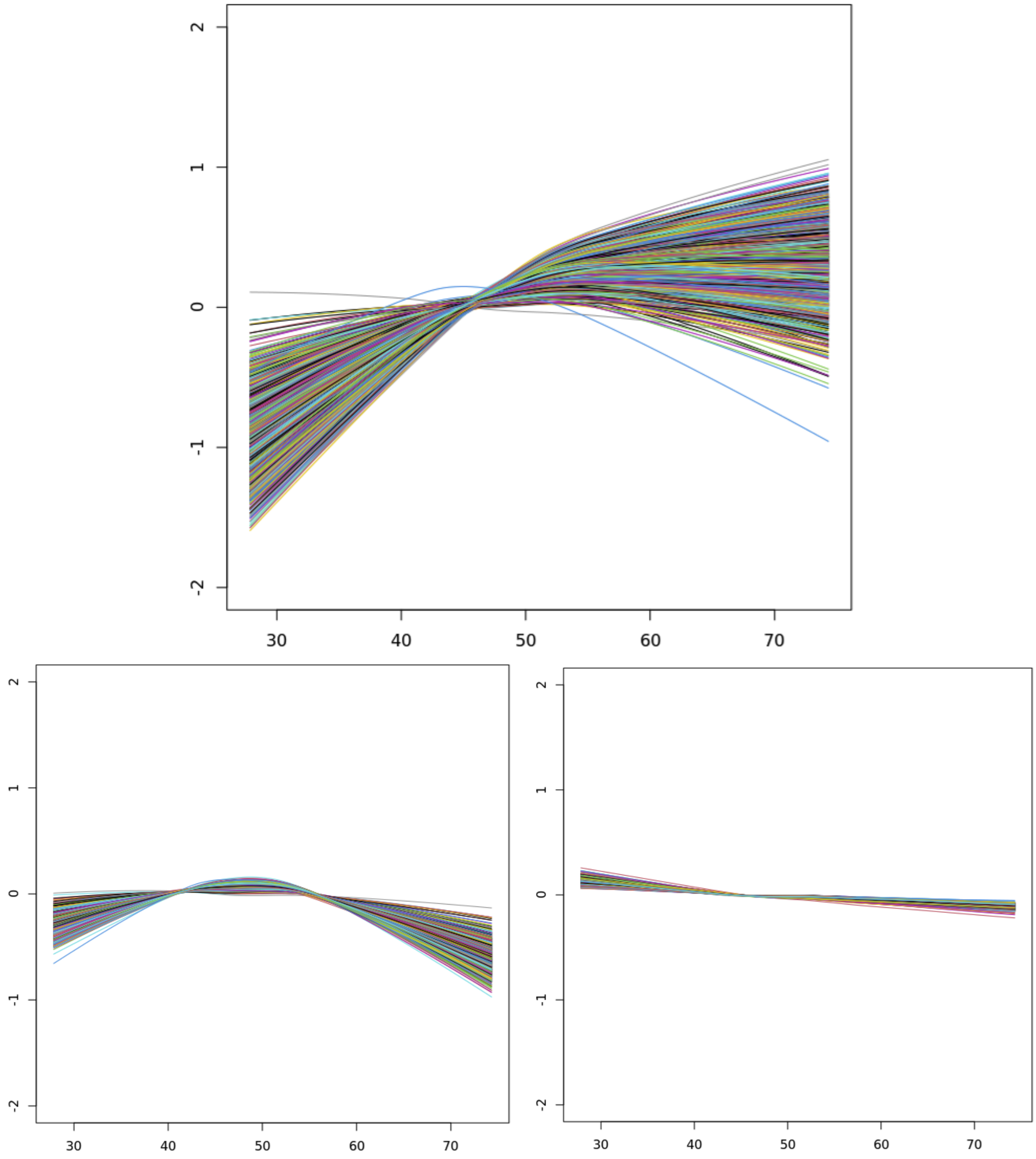


Figure S3: GC content bias correction. Top panel is before correction; Bottom left is after correction with local linear regression; Bottom right is after correction with local quadratic regression (loess in R). Each line represents fitted values of one sample.

## 5 Gene sets for different $\theta$

| Set | eQTL | $D_{TSS}$ | Genic | Intron | Exon | ncRNA | SNP | GWAS |
|---|---|---|---|---|---|---|---|---|
| $S_O$ | 713 | **666.9** | 0.293 | **0.630** | 0.006 | 0.020 | 505 | **0.022** |
| $S_P$ | 10,113 | 115.3 | 0.354 | 0.490 | 0.015 | 0.088 | 9,276 | **0.069** |
| $S_M$ | 9,816 | 135.3 | 0.373 | 0.481 | 0.013 | 0.075 | 8,666 | 0.050 |
| $S_G$ | 895,880 | 84.4 | 0.366 | 0.482 | 0.016 | 0.077 | 584,449 | 0.054 |
| $S_O$ | 688 | **673.2** | 0.260 | **0.663** | 0.006 | 0.026 | 485 | **0.023** |
| $S_P$ | 15,576 | 122.9 | 0.362 | 0.490 | 0.016 | 0.080 | 14,372 | **0.066** |
| $S_M$ | 14,783 | 122.5 | 0.365 | 0.487 | 0.016 | 0.076 | 13,293 | 0.050 |
| $S_G$ | 884,119 | 84.1 | 0.383 | 0.449 | 0.017 | 0.091 | 577,701 | 0.054 |
| $S_O$ | 664 | **671.7** | 0.255 | **0.670** | 0.005 | 0.026 | 474 | **0.023** |
| $S_P$ | 19,288 | 120.1 | 0.371 | 0.483 | 0.016 | 0.079 | 17,701 | **0.063** |
| $S_M$ | 18,311 | 119.5 | 0.376 | 0.475 | 0.016 | 0.075 | 16,544 | 0.050 |
| $S_G$ | 872,406 | 83.8 | 0.382 | 0.448 | 0.017 | 0.091 | 571,184 | 0.054 |

Table S2: Annotation of eQTL for different threshold. Top tabular: $\theta = 0$. Middle tabular $\theta = \log_{10} 2$. Bottom tabular: threshold= $\theta = \log_{10} 3$. $S_O$ is a set of opposing eQTL, $S_P$ is a set of paternal eQTL, $S_M$ is a set of maternal eQTL, and $S_G$ is a set of genotype eQTL. The column eQTL contain counts of eQTL in each set, and column SNP contains counts of distinct SNPs of eQTL in the set. The column *eGene* is the number of genes associated with eQTL in the set. The column of Len is the median length in Kb of the eGenes. (The pattern is the same with the mean length.) The column $D_{TSS}$ contains median distance in Kb to transcription start site. The column GWAS contains percent of GWAS hits among SNPs, with GWAS p-value threshold of $5 \times 10^{-8}$.

# 6 Gene sets

## 6.1 Genes harbor exclusively paternal eQTL $G_1$

ANGEL1, ANKRD11, ANKS6, AQP3, ARNILA, ASB3, BAG3, BCL2L12, C21orf58, C2orf49-DT, CA11, CAMSAP2, CASP9, CCDC96, CCR9, CDKL3, CEBPZ, CFH, CHKA, CLIP4, CPNE5, CRK, CRY2, DBP, DCANP1, DDX51, DISP2, DLX4, EEF1E1, EHMT1, EPB41L2, FAM136A, FAM220A, FAM50B, FLT3, GEMIN4, GMCL1, GNA15-DT, GRAMD1A, HCLS1, HINFP, HPS6, HSDL1, KPNA2, LCDR, LINC00656, LINC01145, LINC01624, LINC01694, LINC03034, LRIG1, LTBR, MAST2, MBD1, MCEE, MED24, MEF2C-AS1, MICAL2, MIF, MIR22HG, MOCS2, MPHOSPH10, MPP7, MRPL39, NCEH1, NCOA2, NDN, NOPCHAP1, NOTCH2NLC, NPM2, NR1H2, PABPC1, PARP15, PCMTD1-DT, PEG10, PER3, PIAS3, PLPP6, POLD1, PPP1CB-DT, PPP1R14BP3, PRR12, PTGS2, PTK2, RBKS, RNF115, RPL5P1, RRM2, SAGSIN1, SCAF1, SCFD1, SEC14L1P1, SGCE, SLC7A5, SLC9A7P1, SMG1P2, SNRPN, SNURF, SQSTM1, TAF6, TANGO2, TARM1, TCAP, TCEA1P2, TIA1, TIMM23B, TINCR, TMEM129, TMEM30A, TMOD1, TRMT2A, TXNIP, UBR5-DT, UROD, VASH1-AS1, VPS9D1-AS1, WAPL-DT, YWHAZ, ZKSCAN5, ZNF276, ZNF34, ZNF407-AS1, ZNF658B, ZNF706, ZNF778-DT, ZNNT1, ZRANB3, ZSCAN16, ZSCAN25

## 6.2 Genes harbor exclusively maternal eQTL $G_0$

ACTBP11, AIP, AKAP8, AMDHD2, ANKRA2, ANKUB1, ANO10, ASAP3, ASCC3, ATG12, BBIP1, BISPR, BLOC1S4, CABP4, CAMKMT, CASS4, CBR3-AS1, CC2D1A, CCDC117, CCDC30, CCT6P1, CDK2AP2, CDKN2AIPNL, CEPT1, CES4A, CMTM3, COA8, CTBP1-DT, CYP2U1, CYSTM1, DDX39A, EIF4B, ELOCP19, EMB, EPS15, EZR, FASTKD3, FEM1A, FUT8-AS1, GALNT4, GLUL, GNL2, GPR108, GRB10, GRK2, GTF2F1, GUCY1B1, GUSB, HAVCR1, HEATR1, HELZ2, HNRNPA1P21, HSPA1A, IGHG4, IGKV2-28, IL23A, ING1, INPP5A, KCTD15, KDM2A, KHSRP, KIR2DL4, LINC00310, LINC00467, LINC02908, LONP1, LRRC8C-DT, LTC4S, LYSMD2, MAN2B2, MAPK8, MCM4, MEG3, METTL15, METTL9, MRFAP1L1, MRPL14, NCF2, NEURL1, NIPSNAP1, NOC4L, NTNG2, NUDT22, OSBPL9, PCM1, PF4, PGGT1B, PI4KAP1, PITPNM1, PLIN3, PMPCB, POC1B-AS1, PPP4R2, PRR5L, PSMB6, PSPN, RANBP3, RASL10A, RCE1, RFX1, RNF43, RPL37AP1, RPS6KB2, RTL10, S1PR2, SCARNA16, SEC23B, SH2D2A, SMIM19, SNHG22, SPRYD7, STAM-DT, STPG3-AS1, SWSAP1, TAGAP, TDP2, TMEM106C, TMEM134, TMEM218, TRAJ37, TRAJ39, TRBV15, UBE2B, UHRF1, USP47, VAV1, VDR, ZBED3-AS1, ZFP37, ZNF286A, ZNF326, ZNF331, ZNF468, ZNF561, ZNF714, ZNF781, ZNF879, ZNF888, ZSWIM4

## 6.3 Genes harbor exclusively opposing eQTL $G_2$

ADAMTSL4-AS1, AFF1, AKR7A2, ANLN, ARID4A, BICD2, BNIP3, BORCS6, BROX, C18orf25, C1orf56, CACNA2D3, CCP110, CD300LF, CD46, CD82, CDK5RAP3, CDKN2A, CENPM, CHD3, CNTROB, COQ7, CPEB4, CPPED1, CXXC5, DNAJB6P1, DPH5-DT,

EBLN2, ECSIT, EXOC5, FAM192BP, FBXW7, FGFR1OP2, FXR2, GABARAP, GNB4, GUCY2D, HEBP1, HSCB, IDI1, IQCK, ITPR1, ITPR2, KDM4A, KDM7A, KIAA0586, LEP, LINC01786, LRRC8D-DT, LZIC, MALINC1, MED21, MIR29B2CHG, MPHOSPH8, MRPS14, MZB1, MZT1, NECAB3, NFIA, NNT-AS1, NOTCH3, OPN3, PALLD, PAPOLG, PARPBP, PEX13, PHIP, PIBF1, PIK3CA, PLSCR3, PODNL1, PPP1R3E, PSMA3-AS1, PSMD9, PSTPIP2, PTRHD1, RARA-AS1, RBL2, REL, RNPC3, RPGRIP1L, RPL23P2, RPS28P7, RYBP, SEC61G, SIGLEC15, SLC15A4, STAM, SYNGR2, TMEM182, TMEM40, TOMM6, TPRG1L, UNC45A, WASL, WRAP53, XPO1, ZBTB4, ZDHHC20

## 6.4 58 phosphoprotein genes

CCP110, ARID4A, WASL, CHD3, BICD2, AFF1, PSMD9, AKR7A2, XPO1, PSTPIP2, SEC61G, PAPOLG, FGFR1OP2, BORCS6, CDK5RAP3, UNC45A, FBXW7, CACNA2D3, TMEM40, RBL2, PLSCR3, PALLD, PPP1R3E, PHIP, EXOC5, CD46, KDM7A, NOTCH3, NECAB3, ZDHHC20, TPRG1L, C1ORF56, ITPR1, ITPR2, RNPC3, CXXC5, ZBTB4, FXR2, SYNGR2, CD300LF, SLC15A4, KDM4A, OPN3, CDKN2A, BNIP3, STAM, PEX13, CPPED1, ANLN, RYBP, NFIA, KIAA0586, REL, GNB4, CNTROB, MPHOSPH8, CPEB4, WRAP53
